# Comparative Transcriptome Profiling of Virulent and Attenuated *Ehrlichia ruminantium* Strains Highlighted Strong Regulation of *map1*- and Metabolism Related Genes

**DOI:** 10.3389/fcimb.2018.00153

**Published:** 2018-05-15

**Authors:** Ludovic Pruneau, Kevin Lebrigand, Bernard Mari, Thierry Lefrançois, Damien F. Meyer, Nathalie Vachiery

**Affiliations:** ^1^CIRAD, UMR ASTRE, Guadeloupe, France; ^2^ASTRE, CIRAD, INRA, University of Montpellier, Montpellier, France; ^3^Université des Antilles, Guadeloupe, France; ^4^Centre National de la Recherche Scientifique, IPMC, Université Côte d'Azur, Valbonne, France; ^5^CIRAD, UMR ASTRE, Montpellier, France

**Keywords:** *Ehrlichia*, transcriptome, pathogenicity, attenuation, virulence

## Abstract

The obligate intracellular pathogenic bacterium, *Ehrlichia ruminantium*, is the causal agent of heartwater, a fatal disease in ruminants transmitted by *Amblyomma* ticks. So far, three strains have been attenuated by successive passages in mammalian cells. The attenuated strains have improved capacity for growth *in vitro*, whereas they induced limited clinical signs *in vivo* and conferred strong protection against homologous challenge. However, the mechanisms of pathogenesis and attenuation remain unknown. In order to improve knowledge of *E. ruminantium* pathogenesis, we performed a comparative transcriptomic analysis of two distant strains of *E. ruminantium*, Gardel and Senegal, and their corresponding attenuated strains. Overall, our results showed an upregulation of gene expression encoding for the metabolism pathway in the attenuated strains compared to the virulent strains, which can probably be associated with higher *in vitro* replicative activity and a better fitness to the host cells. We also observed a significant differential expression of membrane protein-encoding genes between the virulent and attenuated strains. A major downregulation of *map1*-related genes was observed for the two attenuated strains, whereas upregulation of genes encoding for hypothetical membrane proteins was observed for the four strains. Moreover, CDS_05140, which encodes for a putative porin, displays the highest gene expression in both attenuated strains. For the attenuated strains, the significant downregulation of *map1*-related gene expression and upregulation of genes encoding other membrane proteins could be important in the implementation of efficient immune responses after vaccination with attenuated vaccines. Moreover, this study revealed an upregulation of gene expression for 8 genes encoding components of Type IV secretion system and 3 potential effectors, mainly in the virulent Gardel strain. Our transcriptomic study, supported by previous proteomic studies, provides and also confirms new information regarding the characterization of genes involved in *E. ruminantium* virulence and attenuation mechanisms.

## Introduction

*Ehrlichia ruminantium* is the causal agent of heartwater, a fatal disease in wild and domestic ruminants (Allsopp, 2010). This disease represents a serious problem for livestock production in endemic areas such as sub-Saharan Africa and the Caribbean, threatening the American continent where indigenous competent ticks are present (Barré et al., 1987; Gondard et al., 2017). Heartwater is a major obstacle to large-scale production of ruminants in Africa, inducing high mortality within herds with susceptible animals. The economic impact has been evaluated at US $5.6 million per year for Zimbabwe and US $44.7 million per year for the SADC region (Southern Africa Development Community) (Mukhebi et al., 1999). Moreover, the US Department of Homeland Security has listed heartwater in the top-12 priority trans-boundary animal diseases. *E. ruminantium* belongs to the order Rickettsiales in the Anaplasmataceae family, which contains other genera such as *Anaplasma* and *Neorickettsia* as well as *Ehrlichia*. It is transmitted by at least ten species of *Amblyomma* ticks, mainly *A. variegatum*, present in Africa and the Caribbean islands, and *A. hebraeum*, present in Southern Africa (Allsopp, 2010).

Over the last 10 years, the development of *in vitro* models and significant technical progress in molecular biology using high throughput integrative methods have provided new knowledge on the global gene expression of Rickettsiales members and particularly on their pathogenesis. For example, transcriptomic studies have been conducted for some Rickettsiales members such as *Anaplasma phagocytophilum, Ehrlichia chaffeensis*, and *Rickettsia prowazekii* (Pruneau et al., 2014). These various transcriptomic studies of bacteria infecting its host or vector revealed the importance of groups of genes involved in Rickettsiales pathogenesis such as genes encoding for outer membrane proteins (OMPs), known to be involved in the interactions of pathogens with host and vector cells, the Type IV secretion system (T4SS) involved in delivering virulence factors inside infected cells, and the establishment of mechanisms for survival and development in the host or vector cells, such as escape in response to osmotic and oxidative stress (Moumène and Meyer, 2015).

In a preliminary transcriptomic study of *E. ruminantium*, the global transcriptome of the virulent Gardel strain (isolated in the Caribbean island of Guadeloupe) was compared during its developmental cycle. There are two main stages: the extracellular and infectious form (elementary body, or EB) and the vegetative intracellular non-infectious form (reticulate body, or RB) (Pruneau et al., 2012). A total of 54 genes showed a differential expression between the RB and EB stages. Several genes involved in metabolism, nutrient exchange, and defense mechanisms, including those involved in resistance to oxidative stress, were significantly induced in RB. This is consistent with the oxidative stress condition and nutrient starvation that seem to occur in *Ehrlichia*-containing vacuoles allowing their survival in this environment. During the EB stage, we showed the expression of the RNA polymerase-binding transcription factor *dksA*, which is also known to induce virulence in other pathogens. Our results suggested a possible role of these genes in promoting *E. ruminantium* development and pathogenicity (Pruneau et al., 2012).

In obligate intracellular bacteria, differential transcriptional and proteomic analysis of OMPs and secreted proteins between the virulent and attenuated strains is crucial to better understand their essential functions, such as host cell invasion, escape from the phagolysosome, intracellular motility and manipulation of the host response to infection.

The *E. ruminantium* genome contains several genes encoding for the Major antigenic protein 1 (Map1) family proteins, a family of OMPs. Previously, a study on the Map1 cluster showed that it was differentially expressed depending on the host and tick cell lines and *E. ruminantium* strains (Bekker et al., 2005). Even if the role of these proteins remains unclear in the pathogenesis, they are highly immunogenic, inducing a strong Map1 antibody response, which so far has not been associated to protection (Marcelino et al., 2007).

A comparative proteomic study between the virulent and attenuated Gardel strains at EB stage (Marcelino et al., 2015) was recently performed. This study revealed an upregulation of proteins involved in virulence, such as Ldp, AnkA, VirB9 and VirB10 for the virulent Gardel, and an upregulation of proteins involved in cell division, metabolism and transport and protein processing for the attenuated Gardel strain. For the virulent Gardel strain, there was also an upregulation of Map1-family proteins, Map1-2, Map1-3, Map1-4, Map1-8, and exclusive detection of Map1-12, whereas Map1-1 was upregulated in the attenuated Gardel. There was also a strong upregulation of a putative porin CDS_05140 with detection of four proteoforms in the attenuated Gardel strain (Marcelino et al., 2015).

To identify potential pathogenesis and mechanisms of attenuation, we investigated the global gene expression profiling of the virulent and attenuated strains of both Gardel and Senegal at infectious EB stage.

Both Senegal and Gardel *E. ruminantium* attenuated strains induced limited clinical signs after intravenous inoculation to naïve animals and conferred good protection against homologous and heterologous challenges (Faburay et al., 2007; Marcelino et al., 2015). They are still able to grow *in vitro* in host endothelial cells and induce cell lysis. However, Marcelino et al showed the reduction of the Gardel growth cycle *in vitro* for the attenuated strain, with cell lysis developing in 4 days compared to 5 days for the virulent one (Marcelino et al., 2015). The Gardel and Senegal strains are phylogenetically distant (Raliniaina et al., 2010; Pilet et al., 2012; Cangi et al., 2016) and need a different number of *in vitro* passages to be attenuated. Gardel is attenuated *in vitro* after ~200 passages in bovine aortic endothelial cells (Marcelino et al., 2015), whereas only ~15 passages were sufficient to attenuate the Senegal strain in bovine umbilical endothelial cells (Jongejan, 1991). Moreover, in terms of vaccine protection, there is no cross-protection between the Gardel and Senegal strains, underlining the presence of polymorphic protective antigens (D. Martinez, personal communication).

In our study, we identified a set of differentially expressed transcripts coding for Map1-family proteins in particular, upregulated in both virulent strains, as well as for hypothetical membrane proteins probably inducing significant modification of membrane organization. Moreover, we showed an upregulation of 8 genes encoding T4SS components and 3 genes encoding for potential effectors only in the virulent Gardel strain, probably associated with virulence mechanisms.

Furthermore, the proportion of gene upregulation related to metabolism and energy production and conversion was higher in both attenuated strains than in the virulent strains. This result reflects a better fitness to cell culture conditions in the attenuated strains, as evidenced by a shorter bacteria growth cycle.

## Materials and methods

### Production of biological samples

Three biological replicates for each strain were used for the microarrays experiments. For virulent and attenuated Gardel, biological samples were produced at passage 38, 43, and 44 (Gp38, 43, and 44) and at passage 238, 243 and 251 (Gp238, 243, and 251) respectively. For virulent Senegal, the replicates were Senegal passage 7 (Sp7) and two passage 8 replicates (Sp8a and Sp8b). For attenuated Senegal, biological samples were Senegal passage 70, 75, and 77 (Sp70, 75, and 77). All the biological samples were produced in bovine aorta endothelial cells as previously described by Emboulé et al. (2009).

After infection with an appropriate inoculum, all cells were infected and the development of *E. ruminantium* growth was observed daily by light microscopy with detection of morula in each cell. *E. ruminantium* cell cultures used to produce biological samples were thus synchronized. When complete cell lysis was confirmed by light microscopy, samples containing supernatant and cellular debris were harvested and then ultra-centrifuged at 20,000 × g for 15 min at 4°C to collect EBs. The pellets were placed in sterile eppendorfs and homogenized in 2.5 mL of TRIzol reagent (Invitrogen). The samples were immediately stored at −80°C before RNA extraction.

It is noteworthy that the duration between cell infection and lysis was 4 days for the Gardel and Senegal attenuated strains and 5 and 6 days for the Gardel and Senegal virulent strains respectively (data not shown). The duration of lysis remained the same within biological replicates.

### Extraction of total RNA and reverse transcription of RNA samples

The total RNA extraction procedure was carried out as described in Emboulé et al. (2009). Total RNA quantification was performed by Nanodrop 2000c (Thermo Scientific), and total RNA samples were pooled in RNase-free water at a final concentration of 0.5 μg/μL for further analysis.

*E. ruminantium* genomic DNA (gDNA) contaminant was evaluated in each RNA sample by performing the *pCS20* PCR using AB128-AB129 primers as described previously with a limited number of cycles (25 cycles) (Martinez et al., 2004). In the RNA samples, no signal was obtained by the *pCS20* PCR (data not shown), indicating limited *E. ruminantium* gDNA contaminant. Afterward, the RNA samples were reverse transcribed by random priming with Superscript II (Invitrogen) according to the manufacturer's instructions. Reverse transcription and PCR amplification of corresponding cDNA were done using *Kpn*I-primers as previously described (Emboulé et al., 2009).

Moreover, the efficiency of reverse transcription was checked using real-time PCR (qPCR) targeting the *E. ruminantium* 16S rRNA gene by processing the RNA and cDNA samples simultaneously. The primers used for qPCR were 16S-F: 5′ AGCGCAACCCTCATCCTTAG 3′ and 16S-R: 5′ AGCCCACCCTATAAGGGCC 3′. The final concentration of these primers was 900nM. SyberGreen Master Mix was used according to the manufacturer's instructions (Applied Biosystems, France). The PCR program was 10 min at 95°C and 45 cycles with 30 s at 95°C, 30 s at 60°C and 1 min at 72°C. The difference of Ct between the RNA and cDNA samples was always higher than 5 cycles, indicating low contamination by gDNA in the RNA samples (data not shown).

### Design of *E. ruminantium* microarrays

*E. ruminantium* microarrays (8 x 60 k) used in this study were manufactured by Agilent, France. The probes were designed with a modified version of the Oligoarray program (Rouillard et al., 2003). Probe design was carried out using the genomes of the three published strains of *E. ruminantium* (virulent Gardel, Welgevonden Erwe and Erwo) (Collins et al., 2005; Frutos et al., 2006) and using the genomes of the three newly sequenced but not yet published strains of *E. ruminantium* (attenuated Gardel, virulent and attenuated Senegal). There were probes common to the six strains and probes specific to one or more strains. Specific probes were designed for virulent and attenuated strains due to differences observed between genome sequences of virulent and attenuated strains (unpublished data). For data analyses, only probes that are specific for Senegal and Gardel strains were used.

The microarrays contained a total of 16,199 probes (60-mer), with three replicates per probe, including 83 bovine genes as negative controls. The selection of *E. ruminantium* probes gave 14,747 specific probes (91%), with Tm between 80 and 92°C containing 30 to 50% GC. These rules were progressively relaxed to get the additional 964 probes for a subset of missing sequences (Tm between 70 and 92°C and a GC % between 20 and 60). Analysis of specificity indicated that 46,696 oligos had 100% identity on 60 bases, 73,513 had 95% identity on 60 bases and 84,767 had 95% identity on at least 50 bases. Cross-reactivity of probes between the strains was assessed using this latter criterion (95% identity on at least 50 bases).

Forty-eight percent of ORF were covered by 10 or more probes, and 90% were covered by 3 or more probes. Experimental data and associated microarray design have been deposited in the NCBI Gene Expression Omnibus (GEO) http://www.ncbi.nlm.nih.gov/geo/ under series GSE55726 and platforms GPL18397.

### Microarrays: cDNA labeling, scanning and data analyses

Two hundreds nanograms of cDNA were randomly amplified and labeled with Cy3-dUTP using the SureTag DNA Complete Labeling Kit (Invitrogen, France). Following purification with reaction purification columns (Invitrogen, France), quantification of Cy3-dUTP incorporation was performed by absorbance measurement at 550 nm. The yield of cDNA labeling ranged between 3 and 5 μg, and the specific activity ranged between 20 and 25 pmol per μg of cDNA, according to the manufacturer's instructions (Invitrogen, France). Microarrays were incubated for 24 h at 65°C, with a rotation of 20 rpm, in a hybridization chamber. After hybridization, stringent washings were performed according to the manufacturer's instructions (Invitrogen, France). Arrays were scanned using an Agilent C microarray scanner and extracted with the Agilent Feature Extraction program (version 10.10). The obtained images were saved in TIFF format and data with the signal intensities of all spots on each image were saved as “.txt” files for further analysis.

Normalization of microarray data was performed using the Limma package (Smyth, 2004) in R (R Core Team, 2013), available from Bioconductor (http://www.bioconductor.org). Inter-slide normalization was performed using the quantile method. Mean log_2_ fold change (FC) between the virulent and attenuated strains was calculated for each probe and a *B* test was performed. Probes that had low or no signals were excluded from the analysis. For each probe, the expression values of the biological replicates for the virulent and attenuated strains were compiled. The average FC per probe was calculated by taking the average of the log_2_ values of the virulent strain from the average of the log_2_ values from the attenuated strain. The statistical significance of differential expression for each probe was calculated using a *t*-test in R 3.2.3 (R Core Team, 2013), and FDR correction was applied using the Benjamini-Hochberg (Benjamini and Hochberg, 1995) method in R.

Moreover, we checked the homogeneity of log_2_-transformed fluorescence intensities between biological replicates by computing linear correlation coefficients. For the virulent and attenuated Gardel and Senegal strains, linear correlation coefficients were >0.6. Accordingly, the mean intensity values of biological replicates could be calculated.

For genes covered by multiple probes, transcripts were considered differentially expressed between the virulent and attenuated strains for Gardel and Senegal when more than 50% of probes mapping the transcripts had an absolute value of log_2_-fold change (FC) ≥ 1 and a *p*-value < 0.1. A mean FC per CDS was then calculated.

The annotations of genes differentially expressed were checked on NCBI. For genes encoding for hypothetical functions, protein patterns were searched in the InterProt database (Quevillon et al., 2005) and alignments were performed using the Basic Local Alignment Search Tool (http://blast.ncbi.nlm.nih.gov/) in order to identify potential functional domains.

### Quantitative reverse transcription PCR for *map1*-related genes

To validate the differential expression of *map1*-related genes, quantitative reverse transcription PCRs (qRTPCR) were done targeting 7 *map1*-related genes on Gp43 vs. Gp243 and Sp7 vs. Sp77. Briefly for each sample, 1.5 μg of RNA were reverse transcribed with the Superscript VILO cDNA synthesis kit (Invitrogen) according to manufacturer's instructions. The quantity of resulting cDNA was determined by qPCR as previously described (Pruneau et al., 2012) using the primers listed in Table S1.

The Fold Change (FC) between virulent and attenuated strains was measured as follows:

R = (number of cDNA for virulent strain) / (number of cDNA for attenuated strain).

The results were expressed in log_2_ FC between virulent and attenuated strains: comparing Gp43 vs. Gp243 and Sp7 vs. Sp77.

## Results

### Transcriptome profile reproducibility

**Figure 1:**
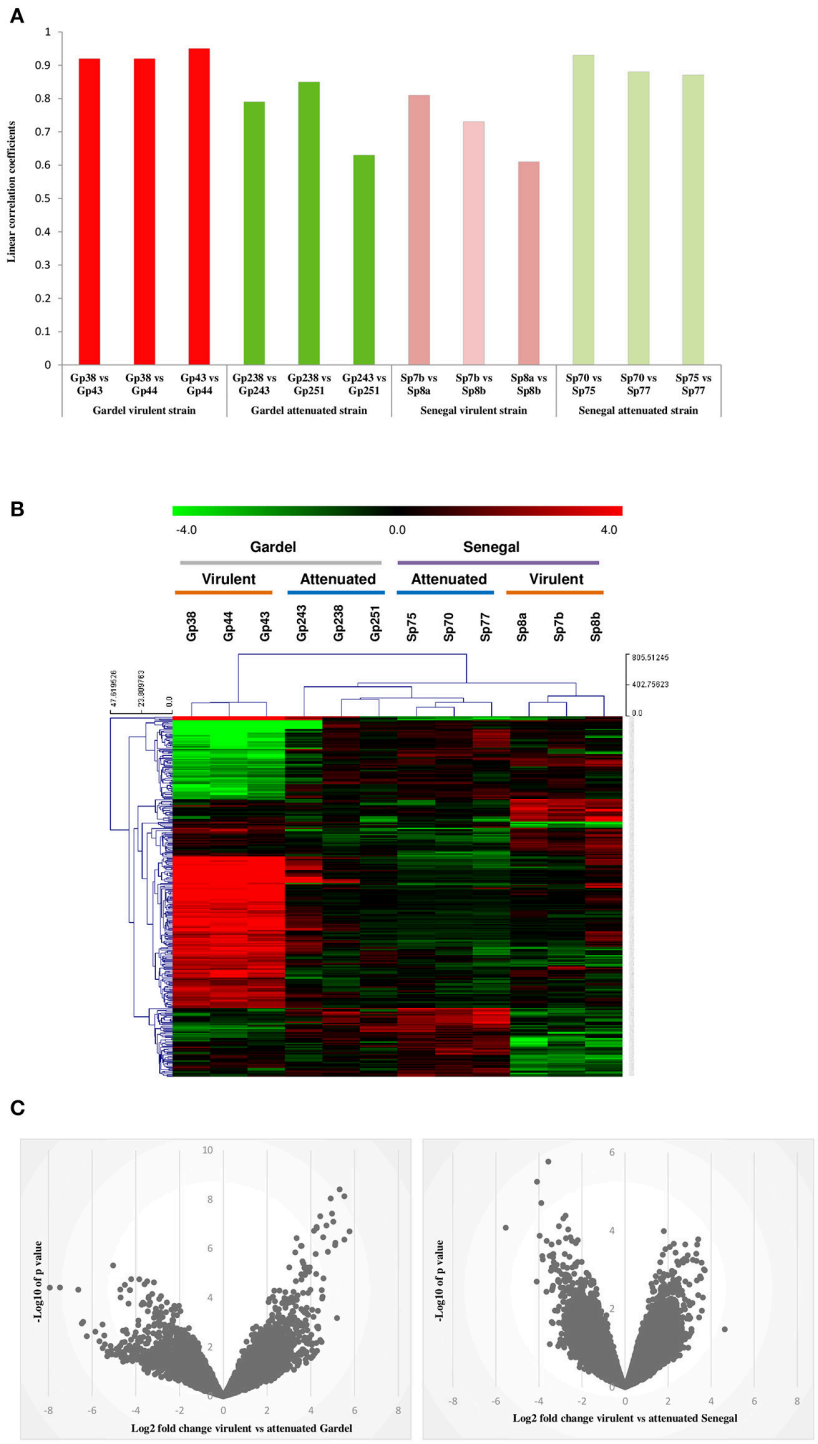
Transcriptome profile reproducibility. **(A)** Linear correlation between the intensity values obtained from different biological replicates for each strain: virulent Senegal (Sp7b, Sp8a and b), attenuated Senegal (Sp70, p75, and 77), virulent Gardel (Gp38, p43, and p44), attenuated Gardel (Gp238, p243, and p251). **(B)** Heatplot showing the expression profile of the 300 most discriminating probes for the virulent and attenuated strains in each sample. Expression corresponds to log_2_ intensity followed by median centering, as visualized on the green to red gradient. Clustering was performed using a Manhattan distance metric and average linkage. **(C)** Volcano plots showing the global transcriptional changes in the virulent vs. attenuated Gardel and Senegal strains. All probes present on the microarray are plotted. Each circle represents one probe. The log_2_ FC is shown on the x-axis. The y-axis shows the log10 of the *p*-value.

Global gene expression profiling of both virulent and attenuated Gardel and Senegal strains was performed using a home-made microarray containing more than 16,000 distinct probes covering all *E. ruminantium* CDS. The mean normalized signal intensities obtained were compared between the different biological replicates for each strain and indicated a good technical reproducibility with the linear correlation coefficients included between 0.6 and 0.94 (Figure 1A). Statistical analysis was then performed to identify the genes differentially expressed between the virulent and attenuated strains, and a clustering was then carried out to identify the 300 best probes discriminating between virulent and attenuated strains (Figure 1B). Clusters based on probe intensity allowed four groups to be identified, with each group including biological triplicates of each strain. The virulent Gardel group was clearly separated from the other groups formed by the attenuated Gardel and the attenuated and virulent Senegal strains (Figure 1B). Furthermore, we observed a clear separation between the virulent Senegal and the attenuated strains of both Gardel and Senegal. Volcano plots showing the global transcriptional changes in the virulent vs. attenuated strains were shown in Figure 1C. Globally, log_2_ FCs were higher for Gardel than for Senegal, with stronger *p*-values.

### General overview of differentially expressed genes between virulent and attenuated strains

**Figure 2:**
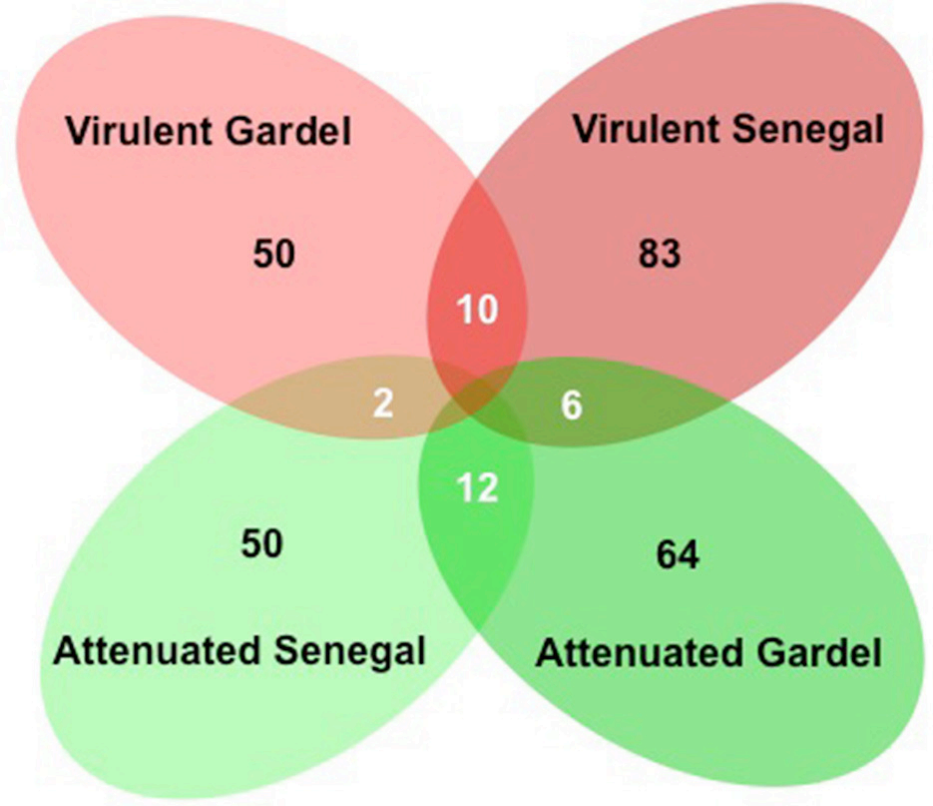
Venn diagram of upregulated genes for the virulent and attenuated strains. Numbers of upregulated genes are indicated for each strain (in black). The numbers of common upregulated genes are indicated in circle intersections (in white).

**Figure 3:**
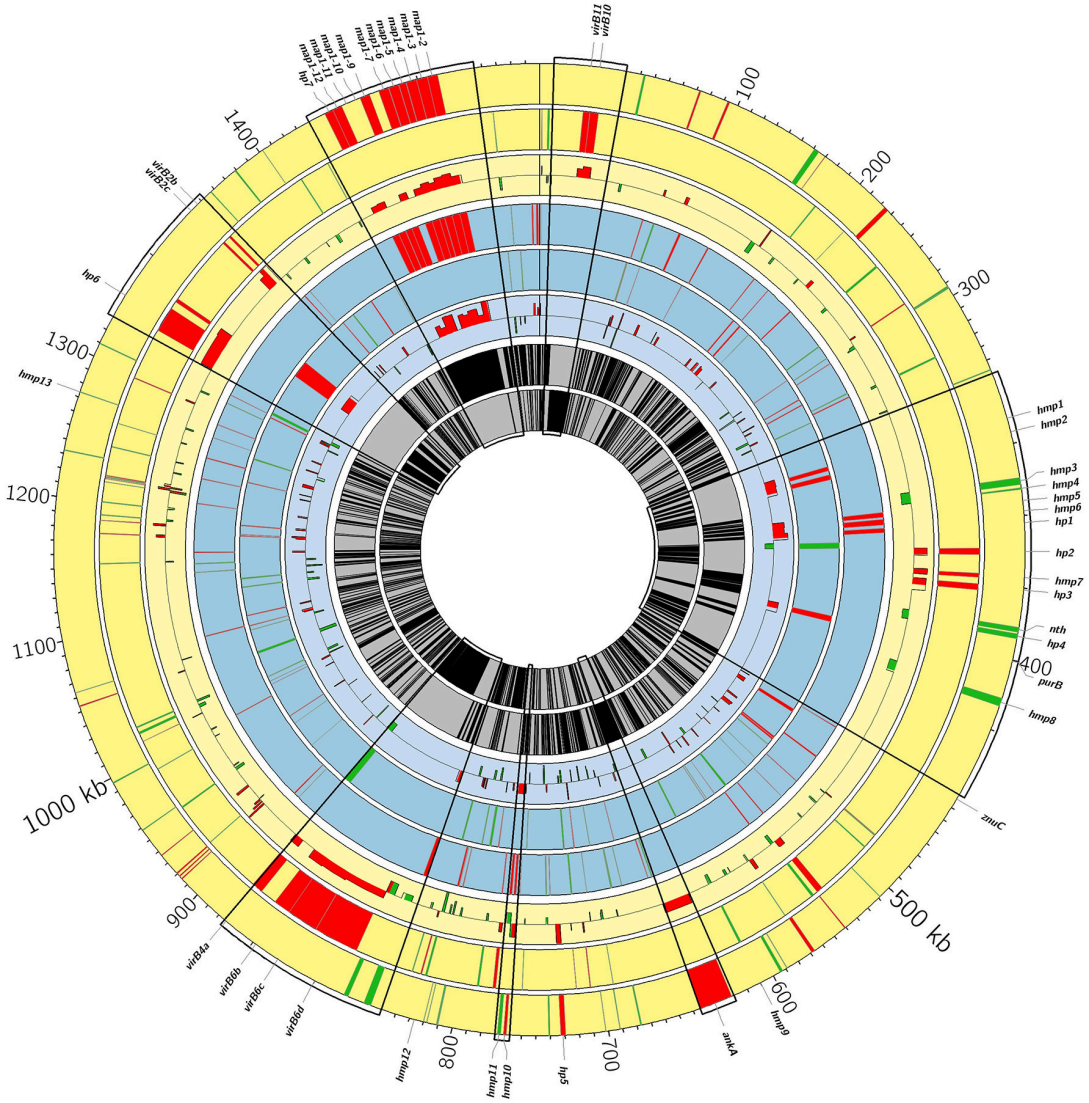
Circos genome diagram showing differentially expressed genes between the virulent and attenuated strains. From outer to inner, positive and negative genome strands for Gardel (yellow) and for Senegal (blue) respectively and mean of log_2_ FC for each CDS are shown. Upregulated genes in the virulent strains are in red and upregulated genes in the attenuated strains are in green. Some areas are enlarged and correspond to *map1*-family genes (magnification 8-fold) and to *Vir* genes and genes encoding hypothetical membrane proteins (hmp) and hypothetical proteins (hp) (magnification 5-fold). Hmp1: CDS 02240; hmp2: CDS 02250; hmp3: CDS 02290; hmp4: CDS 02300; hmp5: CDS 02320; hmp6: CDS 02330; hmp7: CDS 02390; hmp8: CDS 02480; hmp9: CDS 03690; hmp 10: CDS 04750; hmp11: CDS 04760; hmp12: CDS 05140; hmp13: CDS 07940. Hp1: CDS 02340; hp2: CDS2370; hp3: CDS 02410; hp4: CDS 02440; hp5: CDS 04510; hp6: CDS 08310; hp7: CDS 09020.

There were 114 and 133 out of 950 genes (12 and 14% of ORFs) differentially expressed between the virulent and attenuated Gardel and between the virulent and attenuated Senegal strains respectively. Fifty genes were found upregulated for the Gardel virulent strain compared to the attenuated strain, and conversely 64 genes were found upregulated for the attenuated Gardel strain compared to the virulent strain (Table 1, Figure 2). For Senegal, the number of upregulated genes was higher for the virulent strain (83 genes) than for the attenuated strain (50 genes) (Table 2, Figure 2). Common upregulation of expression for 10 genes was observed for both virulent strains and 12 for both attenuated strains (Figure 2, white numbers). The detailed list of upregulated genes for both Gardel and Senegal are shown in Tables 1, 2. Interestingly, the common upregulated genes in the virulent strains included 8 *map-1* genes (*map1-3, map1-2, map1-4, map1-6, map1-9, map1-5, map1-7*, and *map1-12*) (Figure 3). The other transcripts coded for proteins involved in translation, ribosomal structure and biogenesis (*truA*) and for a hypothetical membrane protein (hmp) (CDS_04750: hmp10) (Tables 1, 2, Figure 3). The common upregulated genes for the attenuated strains belong to energy production and conversion (*fdxB* and *atpG*), nucleotide transport and metabolism (*ndk*), replication, recombination and DNA repair (*ssb*), hypothetical membrane proteins (CDS_03690: hmp9 and CDS_05140: hmp12) and hypothetical proteins (hp) (CDS_09210, CDS_3280, CDS_04190, CDS_08200, CDS_03450 and CDS_05460) (Tables 1, 2).

**Table 1:** Upregulated genes and their function for the Gardel strains (genome accession numbers: NC_006831.1).

**Gene function**	**Virulent**			**Attenuated**		
	**Gene id**	**Name**	**log_2_ FC**	**Gene id**	**Name**	**log_2_ FC**
Energy production and conversion	CDS_05160^c^	*petC*	1.19	**CDS_04300**	***fdxB***	**1.70**
				**CDS_04070**	***atpG***	**1.28**
				CDS_00110	*fdxA*	1.58
				CDS_08460	*ctaG*	1.31
				CDS_08790	*atpE*	1.10
Nucleotide transport and metabolism				**CDS_08980**^a^	***ndk***	**1.17**
				CDS_02940	*dnaX*	1.73
				CDS_07270^a^	*purC*	1.54
				CDS_07770	*tmk*	1.50
				CDS_07540	*pyrH*	1.32
Coenzyme transport and metabolism	**CDS_00530**^c^	***hemA***	**1.21**	**CDS_01790**^c^	***pdxH***	**1.64**
	CDS_01740	*coaX*	1.18	**CDS_07700**	***ribE***	**1.55**
Replication, recombination and DNA repair	CDS_03430^c^	*topA*	2.05	**CDS_02840**^a^	***ssb***	**1.11**
	CDS_00030	*ruvC*	1.75	CDS_02430	*nth*	1.21
	CDS_06660	*radC*	1.37	CDS_07870	*xseB*	1.12
Posttranslational modification, protein turnover, chaperones	CDS_04780	*clpA*	1.75	**CDS_03060**	***ccmE***	**1.14**
				CDS_05010^a^	*bcp*	1.64
Carbohydrate/AA/inorganic ion transport and metabolism	CDS_03250	*gdh2*	1.42	CDS_05860	*thio*	1.92
				CDS_04200	*rpiB*	1.30
Defense mechanisms				CDS_01120	*lolD*	1.52
Lipid transport and metabolism				CDS_02180	*pgpA*	1.62
				CDS_04850	*ispG*	1.42
				CDS_05330	*ispH*	1.14
				CDS_05660		1.13
Cell wall/ membrane/envelope biogenesis				CDS_08860	*lgt*	1.61
				CDS_08610^c^	*dacF*	1.33
				CDS_06490	*aprD*	1.30
				CDS_01510	*aprE*	1.14
				CDS_03590	*lepA*	1.06
Intracellular trafficking, secretion, and vesicular transport	**CDS_05400**	***virB4a***	**1.51**			
	CDS_08380	*virB2b*	3.72			
	CDS_08370	*virB2c*	2.62			
	CDS_00150^b^	*virB10*	2.08			
	CDS_05380	*virB6b*	1.77			
	CDS_05370	*virB6c*	1.59			
	CDS_00140^a^	*virB11*	1.60			
	CDS_05360	*virB6d*	1.31			
Map1-related protein	**CDS_09120**^c^	***map1-3***	**2.36**			
	**CDS_09110**^c^	***map1-4***	**2.14**			
	**CDS_09090**	***map1-6***	**1.84**			
	**CDS_09130**^c^	***map1-2***	**1.71**			
	**CDS_09100**^c^	***map1-5***	**1.44**			
	**CDS_09060**	***map1-9***	**1.36**			
	**CDS_09030**^b^	***map1-12***	**1.29**			
	**CDS_09080**	***map1-7***	**1.28**			
Translation, ribosomal structure and biogenesis	**CDS_04360**^c^	***truA***	**1.94**	**CDS_05070**	***rrmJ***	**2.50**
				CDS_05020	*rsmE*	1.66
				CDS_00280	*trmE*	1,46
				CDS_05240	*rpsB*	1,18
				CDS_06320	*spoU*	1,16
				CDS_03560	*rpsO*	1,10
				CDS_06190^c^	*rplN*	1,00
Hypothetical membrane protein	**CDS_04750**		**2.56**	**CDS_05140**^b^		**3.82**
	CDS_02390		2.62	**CDS_03690**		**1.81**
	CDS_07440		2.41	**CDS_07940**^c^		**1.59**
	CDS_07610		2.29	CDS_06520^c^	*T4SS Putative effector*	2.57
	CDS_06460		2.04	CDS_00970	*T4SS Putative effector*	2.30
	CDS_07370		1.80	CDS_07620		2.22
	CDS_05650^c^		1.40	CDS_02300		1.78
	CDS_03260		1.28	CDS_05100		1.75
	CDS_07630		1.21	CDS_02290		1.71
				CDS_02480		1.41
				CDS_07470		1.25
				CDS_05900		1.23
				CDS_02040		1.12
Hypothetical protein	CDS_01030		3.81	**CDS_09210**^a^		**2.46**
	CDS_05600		3.69	**CDS_03280**		**1.88**
	CDS_04510	*T4SS Putative Effector*	3.57	**CDS_04190**		**1.81**
	CDS_05770^c^		2.76	**CDS_08200**^a^	***LexA***	**1.76**
	CDS_08320		2.51	**CDS_03450**		**1.44**
	CDS_02410		2.51	**CDS_04560**^c^	***sspB***	**1.33**
	CDS_02370^b^		2.49	**CDS_04760**		**1.99**
	CDS_08310^a^	*T4SS Putative Effector*	2.31	CDS_07600		3.10
	CDS_05620		2.18	CDS_05350		2.24
	CDS_08100		1.82	CDS_06720		1.98
	CDS_03830^b^	*AnkA*	1.78	CDS_00080		1.45
	CDS_01350		1.56	CDS_01260		1.25
	CDS_00600		1.43	CDS_04680^c^		1.10
	CDS_02830		1.50	CDS_02440^a^		1.10
	CDS_04430		1.38	CDS_01780^c^		1.05
	CDS_09020		1.16			
Number of upregulated genes			50			64

*Bold blue: common upregulated genes in the Gardel and Senegal strains*.

*Bold pink: conversely upregulated genes in the Gardel and Senegal strains*.

a *gene encoding for proteins detected in a proteomic study comparing the virulent and attenuated Gardel strains (Marcelino et al., 2015)*.

b *gene encoding for proteins differentially expressed in a proteomic study comparing the virulent and attenuated Gardel strains (Marcelino et al., 2015)*.

c *gene encoding for strain-specific protein based on a proteomic study comparing the virulent and attenuated Gardel strains (Marcelino et al., 2015)*.

**Table 2:** Upregulated genes and their function for the Senegal strains (genome accession numbers: NC_006831.1).

**Gene function**	**Virulent**			**Attenuated**		
	**Gene id**	**Name**	**log_2_ FC**	**Gene id**	**Name**	**log_2_ FC**
Energy production and conversion	CDS_04540	*nuoE*	1.16	**CDS_04300**	***fdxB***	**1.76**
				**CDS_04070**	***atpG***	**1.41**
				CDS_04900	*nuoL*	1.90
				CDS_04410	*nuoH*	1.87
				CDS_06480	*qor2*	1.60
				CDS_04700	*atpC*	1.59
				CDS_08270	*acnA*	1.26
				CDS_03100	*nuoA*	1.10
				CDS_04910	*nuoK*	1.09
Nucleotide transport and metabolism	CDS_06810	*purM*	1.56	**CDS_08980**	***ndk***	**1.70**
	CDS_02460	*purB*	1.50	CDS_07060	*thyX*	1.72
	CDS_06330	*cmk*	1.33			
Coenzyme transport and metabolism	**CDS_07700**	***ribE***	**1.57**	**CDS_00530**	***hemA***	**2.87**
	**CDS_01790**	***pdxH***	**1.37**	CDS_04080	*folE*	1.89
	CDS_09450	*ubiF*	1.72	CDS_01680	*bioF*	1.58
	CDS_06870	*gshB*	1.63			
	CDS_08040	*ubiE*	1.52			
	CDS_02030	*thiE*	1.25			
	CDS_02910	*nadD*	1.06			
Replication, recombination and DNA repair	CDS_01880	*dnaA*	1.90	**CDS_02840**	***ssb***	**1.24**
	CDS_05050	*recO*	1.53			
	CDS_01110	*xerD*	1.32			
	CDS_00800	*holA*	1.31			
Posttranslational modification, protein turnover, chaperones	CDS_07310	*coxW*	1.82	CDS_06620	*clpB*	2.44
	**CDS_03060**	***ccmE***	**1.32**	CDS_07200	*grxC2*	1.66
	CDS_05090		1.29	CDS_08630	*dsbD*	1.43
Carbohydrate/AA/inorganic ion transport and metabolism	CDS_02740	*proP*	1.73	CDS_07110	*glyA*	1.03
	CDS_05960	*pstB*	1.73			
	CDS_05320	*carA*	1.66			
	CDS_00460	*rpe*	1.59			
	CDS_08830	*trkH*	1.44			
	CDS_02590	*znuC*	1.42			
	CDS_03380	*cutA*	1.22			
Lipid transport and metabolism	CDS_03460	*acpS*	1.33			
Cell cycle control, cell division, chromosome partitioning	CDS_08240	*parA*	1.48			
Cell wall/ membrane/envelope biogenesis	CDS_01150	*Int*	1.47	CDS_07250	*wcaG*	1.45
				CDS_04980	*mraW*	1.36
Intracellular trafficking, secretion and vesicular transport	CDS_07740	*secB*	1.77	**CDS_05400**	***virB4a***	**1.20**
	CDS_04840	*tatC*	1.48	CDS_05410	*virB3*	2.43
Map1-related protein	**CDS_09130**	***map1-2***	**2.87**			
	**CDS_09060**	***map1-9***	**2.40**			
	**CDS_09100**	***map1-5***	**2.11**			
	**CDS_09110**	***map1-4***	**2.10**			
	**CDS_09080**	***map1-7***	**1.96**			
	**CDS_09090**	***map1-6***	**1.67**			
	**CDS_09030**	***map1-12***	**1.62**			
	**CDS_09120**	***map1-3***	**1.07**			
	CDS_09050	*map1-10*	2.47			
	CDS_09040	*map1-11*	1.10			
Translation, ribosomal structure and biogenesis	**CDS_04360**	***truA***	**2.42**	CDS_05500	*rpmB*	2.42
	**CDS_05070**	***rrmJ***	**1.45**	CDS_01760		2.58
	CDS_03220	*rluC*	1.53	CDS_02000	*fmt*	1.89
	CDS_08540	*hemK*	1.51	CDS_01290	*rplT*	1.51
	CDS_08260		1.42	CDS_09290	*rplS*	1.24
	CDS_00440		1.28	CDS_04050	*raiA*	1.08
	CDS_09490	*glyS*	1.13			
Hypothetical membrane protein	**CDS_07940**		**1.80**	**CDS_03690**		**1.49**
	**CDS_04750**		**1.43**	**CDS_05140**		**1.48**
	CDS_07410		2.00	CDS_07920	*surA*	1.14
	CDS_00760		1.84	CDS_03840		1.11
	CDS_02790		1.69			
	CDS_02320		1.67			
	CDS_02330		1.58			
	CDS_02240		1.51			
	CDS_07580		1.43			
	CDS_02250		1.43			
	CDS_04730		1.37			
	CDS_04790		1.11			
Hypothetical protein	**CDS_04760**		**1.43**	**CDS_04560**	***sspB***	**2.68**
	CDS_00540		2.38	**CDS_09210**		**2.32**
	CDS_02340		2.04	**CDS_03450**		**2.13**
	CDS_05530		1.93	**CDS_04190**		**2.10**
	CDS_00020		1.70	**CDS_08200**	***LexA***	**1.60**
	CDS_03330		1.65	**CDS_03280**		**1.07**
	CDS_07820		1.63	CDS_07190		2.30
	CDS_00620		1.60	CDS_06440		2.18
	CDS_08490		1.59	CDS_00430		1.92
	CDS_02800		1.51	CDS_05720		1.77
	CDS_07490		1.50	CDS_02070		1.56
	CDS_01720		1.50	CDS_05890		1.48
	CDS_01200		1.46	CDS_06760		1.44
	CDS_04010		1.46	CDS_03000		1.29
	CDS_07400		1.40	CDS_02360		1.25
	CDS_06420		1.35	CDS_04440		1.06
	CDS_01390		1.33	CDS_09350		1.06
	CDS_08340		1.31			
	CDS_06800		1.30			
	CDS_03870		1.28			
	CDS_08440		1.25			
	CDS_01210		1.20			
	CDS_02630		1.17			
	CDS_01930		1.11			
Number of upregulated genes			83			50

*Bold blue: common upregulated genes in the Gardel and Senegal strains*.

*Bold pink: conversely upregulated genes in the Gardel and Senegal strains*.

**Figure 4:**
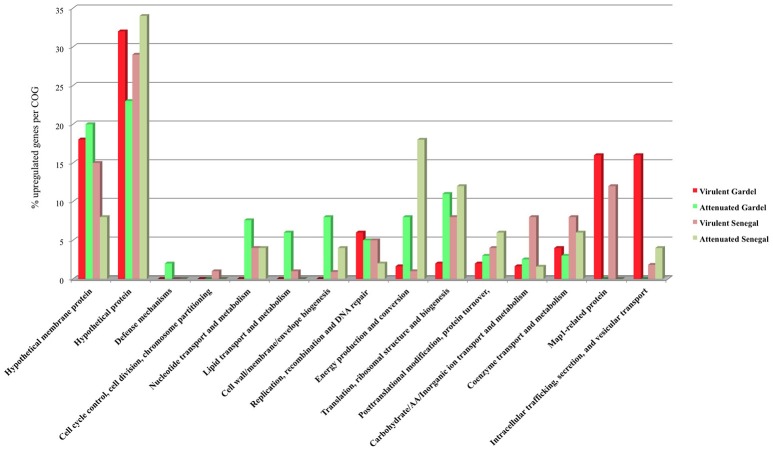
Proportion of upregulated genes per COG for the virulent and attenuated strains.

The upregulated genes were classified according to their COG (clusters of orthologous groups of proteins) function. The proportion of upregulated genes in each COG for the virulent and attenuated Gardel and for the virulent and attenuated Senegal strains is shown in Figure 4. The genes encoding hypothetical proteins represented the highest proportion of upregulated genes with 32 and 23% for the virulent and attenuated Gardel and 29 and 34% for the virulent and attenuated Senegal strains respectively. The second highly upregulated gene COG corresponded to a “hypothetical membrane protein” for the virulent and attenuated Gardel strains. Genes belonging to several COGs were exclusively found to be upregulated only in a single strain: i.e., defense mechanisms upregulated in the attenuated Gardel strain, and cell cycle control, cell division, chromosome partitioning in the virulent Senegal strain (Figure 4). Nonetheless, the proportion of genes upregulated in strain-specific COGs was very low (<2%).

There was upregulation of genes belonging to nucleotide transport and metabolism and cell wall/membrane/envelope biogenesis in both virulent and attenuated Senegal and in the attenuated Gardel strain only (Figure 4).

Genes involved in metabolic functions such as energy production and conversion; translation, ribosomal structure and biogenesis; and posttranslational modification, protein turnover, chaperones were upregulated for both virulent and attenuated strains of Gardel and Senegal, but with more genes upregulated for the attenuated strains overall. Similarly, there was an upregulation for the Gardel and Senegal strains for genes involved in carbohydrate/Amino acid/Inorganic ion transport and metabolism, coenzyme transport and metabolism, but with more genes upregulated in the virulent Senegal strain (Figure 4).

### Upregulation of the *map1* gene family in virulent strains

The percentage of upregulated genes belonging to the *map1*-related gene family was higher and more specific for the virulent strains than for the attenuated strains: 16 and 12% for the virulent Gardel and Senegal strains respectively (Figure 4). Eight out of 16 *map1*-family genes were upregulated in the virulent strains for both Gardel and Senegal as described above (Tables 1, 2, Figure 3). Additionally, two genes were exclusively upregulated in the virulent Senegal strain: *map1-10* and *map1-11* (Figure 3).

Seven *map1*-related genes were chosen for a qPCR validation of the microarray results. The log_2_ FC obtained by qRTPCR for Gp 43 vs. Gp 243 and for Sp7 vs. Sp77 were compared to log_2_ FC obtained by microarrays (Table 3). The upregulation of 6 *map1*-related genes, observed by microarrays was confirmed by qRTPCR for both virulent Gardel and Senegal. For *map1-10* gene expression in Gardel strains, there was no significant differential expression obtained by microarrays which was confirmed by qRTPCR (log_2_ FC = −0.6) but we confirmed the upregulation of *map1-10* exclusively for virulent Senegal strain with a FC = 4.1 obtained by qRTPCR and 2.47 obtained by microarray (Table 3). The *map1-4* qRTPCR could not be optimized for Senegal strain, thus confirmation of microarray result on *map1-4* was not possible.

**Table 3:** Comparison of log_2_ FC for *map1*-related genes obtained by microarrays and qRTPCR.

**Gene name**	**Gene Id**	**Gardel**		**Senegal**	
		**log_2_ FC microarrays**	**log_2_ FC qRTPCR**	**log_2_ FC microarrays**	**log_2_ FC qRTPCR**
*map1-12*	CDS_09030	1.29	1.56	1.62	11.6
*map1-10*	CDS_09050	1^*^	−0.6	2.47	4.1
*map1-9*	CDS_09060	1.36	1.17	2.4	2.2
*map1-6*	CDS_09090	1.84	1.61	1.67	2.2
*map1-4*	CDS_09110	2.14	1.38	2.10	ND
*map1-3*	CDS_09120	2.36	2.57	1.07	3.69
*map1-2*	CDS_09130	1.71	9.27	2.87	2.8

*ND, not detected*.

* *not significantly differentially expressed*.

### Differential expression of genes encoding for hypothetical proteins

Genes encoding for hypothetical proteins (hp), including hypothetical membrane proteins (hmp), were found to be upregulated for both virulent and attenuated strains of Gardel and Senegal (Tables 1, **2**). There were 11 upregulated genes in common between the Gardel and Senegal strains. For hypothetical membrane proteins, CDS_03690 (hmp9) and CDS_05140 (hmp12) were upregulated in the attenuated strains of both Gardel and Senegal, whereas CDS_04750 (hmp10) was upregulated in the virulent strains. CDS_07940 (hmp13) and CDS_04760 (hmp11) were upregulated in attenuated Gardel and conversely upregulated in virulent Senegal (Figure 3). CDS_03690 (hmp9) and CDS_04750 (hmp10) encode for proteins that are unique to *E. ruminantium*. Some genes encoding hypothetical membrane proteins are located in the same chromosomal region and were identically modulated between the virulent and attenuated strains (Figure 3). For example, CDS_02240 (hmp1) and CDS_02250 (hmp2) or CDS_02320 (hmp5), CDS_02330 (hmp6) and CDS_02340 (hp1) were upregulated for virulent Senegal and CDS_02290 (hmp3) and CDS_02300 (hmp4) were upregulated for attenuated Gardel. CDS_02440 (hp4) associated with *nth* (CDS_2430) are also upregulated in attenuated Gardel (Figure 3).

### Differential expression of T4SS components between virulent and attenuated gardel strains

In *E. ruminantium*, genes encoding for T4SS are organized into five clusters comprising two major operons. Eight *vir* genes were upregulated only in virulent Gardel with a higher log_2_-FC ranging from 3.72 to 1.31 (Table 1). There are *virB4a, virB6b, virB6c*, and *virB6d* localized on one operon (operon *virB3-B6*); *virB10* and *virB11* localized on a second operon (operon *virB8-11/D4*); and *virB2b* and *virB2c* localized on a third operon (operon *virB4b/B2a-d*), as shown in Figure 3. Moreover, CDS_04510 (hp5), CDS_08310 (hp6) and CDS_03830 (homolog of *ankA*) have been identified by S4TE software as potential effectors of T4SS and were also upregulated in virulent Gardel (Meyer et al., 2013). CDS_06520 and CDS_00970 are also potential effectors and were upregulated in attenuated Gardel.

For Senegal, there were only 2 *vir* genes (*virB3* and *virB4a*) differentially expressed between the virulent and attenuated strains, but contrary to Gardel, they were upregulated in the attenuated strain (Table 2, Figure 3).

### *E. ruminantium* defense against host cell immune response

Among the genes differentially expressed between the virulent and attenuated Gardel and Senegal strains, several genes are involved in the subversion of host cell response. In fact, *grxC2*, which encodes for glutaredoxin, was upregulated in the attenuated Senegal strain whereas *bcp*, which encodes for the Bacterioferritin co-migratory protein, was upregulated in the attenuated Gardel strain. Only Senegal displayed differential gene expression for genes involved in counteracting osmotic stress. For Senegal, there were 8 genes differentially expressed between the virulent and attenuated strains. Genes encoding for proline-betaine transporter (*proP*), for NADH dehydrogenase subunit E (*nuoE*) and for a putative Na+/H+ antiporter subunit (CDS_01720) were upregulated for the virulent strain (Table 2). Genes *nuoH, nuoL, nuoA*, and *nuoK* encoding for NADH-quinone oxidoreductase and CDS_05720 (Putative Na+/H+ antiporter subunit) were upregulated for the attenuated strain (Table 2).

Other genes known to be induced during stress were found to be differentially expressed between the virulent and attenuated strains with a high log_2_-FC. For example, gene encoding for protein ClpB (2.44) and CDS_07190 (2.30) encoding for BolA-like protein were upregulated in attenuated Senegal (Table 2).

## Discussion

In the Anaplasmataceae family, most studies have been conducted on virulent strains and have identified a small number of key components of pathogenicity (Pruneau et al., 2014). However, there are a few studies on attenuated strains, comparing them with virulent strains in order to identify mechanisms of attenuation. In *E. ruminantium*, three strains have been attenuated *in vitro* by successive passages in canine macrophages for the Welgevonden strain (Zweygarth et al., 2005) and in bovine endothelial cells for the Senegal (Jongejan, 1991) and Gardel strains (Marcelino et al., 2015). These attenuated strains confer strong and long-lasting protection against homologous and some heterologous challenges (Faburay et al., 2007). However, so far, the mechanisms of attenuation remain unknown and the use of attenuated vaccines is limited partly due to the possible reversion of virulence. In this study, to investigate both the virulence and attenuation mechanisms, we compared the global transcriptome profile of two distant virulent strains of *E. ruminantium*, Gardel and Senegal, with their corresponding attenuated strains *in vitro*. Moreover, three biological replicates per strain were used, and the transcriptome profiles obtained between the different replicates were at least 72% identical (main linear correlation coefficient), thus strengthening our microarray data.

Transcriptomic data for the Gardel strains obtained in this study were compared with previous proteomic data obtained by comparing the Gardel virulent and attenuated strains (Marcelino et al., 2015). Among the proteins upregulated or detected only in virulent Gardel in the proteomic study, 10 and 1 corresponding genes were found upregulated in our transcriptomic results for virulent and attenuated Gardel, respectively. From these 10 upregulated genes, there are 4 MAP1-related proteins (*map1-3, map1-4, map1-2*, and *map1-12*) and 2 genes encoding proteins related to virulence (*virB10* and *ankA*) (Table 1). Furthermore, among the proteins upregulated or detected only in attenuated Gardel by the proteomic analysis, 9 corresponding genes were upregulated for the attenuated strain and 4 for the virulent strain. Finally, CDS_05140 (hmp12), which displays the highest value of FC, was the second most abundant protein in the proteomic study (Marcelino et al., 2015). The transcriptomic and proteomic studies are consistent: for the virulent strain, an upregulation of Map1-family and virulence gene and protein expression, and for the attenuated strain, an upregulation of gene and protein expression related to metabolism.

The virulent Gardel strain showed a different transcriptomic profile compared to others strains (Figure 1B). This result correlates with the COG differences observed between the four strains. In fact, two COGs were absent in the virulent Gardel strain when compared to other strains (Nucleotide transport and metabolism; Cell wall/membrane/envelope biogenesis) and three others displayed the highest proportion of upregulated genes (Figure 4). This result suggests that the virulence phenotype may be associated with distinct gene subsets. In fact, these categories of COGs contain genes related to virulence, such as *vir* genes encoding components of T4SS. *Vir* genes upregulated in virulent Gardel encode for two major T4SS clusters (Figure 3). The T4SS is a protein complex that is important for the pathogenesis of intracellular bacteria because it permits translocation of virulence factors into the host cell (Voth et al., 2012). The crucial role of T4SS and its effector proteins such as AnkA has been shown for two others pathogens of the Anaplasmataceae family, *Anaplasma phagocytophilum* and *Ehrlichia chaffeensis* (Rikihisa and Lin, 2010). The upregulation of genes encoding for T4SS together with gene ERGA_CDS_03830, encoding for the AnkA homolog, suggests an important role in the virulence of the virulent Gardel strain of *E. ruminantium*.

Another gene ERGA_CDS_06440, encoding for the AnkB homolog, was found upregulated in attenuated Senegal, as well as two genes encoding for T4SS components (*virB3* and *virB4a*), whereas no upregulation of *vir* genes was observed in the virulent Senegal strain. Moreover, ERGA_CDS_03830 (homolog of *ankA*) and ERGA_CDS_06440 were predicted as potential T4SS effectors for *E. ruminantium* using S4TE software (Meyer et al., 2013). In a newly published transcriptomic study on the virulent Welgevonden strain of *E. ruminantium*, genes encoding for T4SS components and homologs of *ankA* and *ankB* were also upregulated in EBs in comparison to RBs (Tjale et al., 2018). Further studies of these genes and genes encoding for T4SS components are crucial for the understanding of *E. ruminantium* pathogenesis.

In our study, genes related to virulence display a differential expression between the virulent and attenuated Gardel strains, whereas limited differential expression was observed for the Senegal strains. The mechanism of attenuation between Gardel and Senegal is probably different because the speed of attenuation for Senegal is faster than that for Gardel. Some virulence factors could be affected by attenuation mechanisms leading to a lack of virulence.

Moreover, the attenuated strains seem to have a higher metabolic activity than the virulent strains. In fact, the proportion of genes involved in energy production and conversion, posttranslational modification, protein turnover, chaperones and translation, ribosomal structure and biogenesis was higher for the attenuated strains. Genes encoding for proteins of ATP synthase such as *atpC, atpE*, and *atpG* were found upregulated only in the attenuated strains. Also, another gene exclusively upregulated in both attenuated strains, *ndk*, encodes for a nucleoside diphosphate kinase, which catalyzes the formation of nucleoside triphosphate required for DNA synthesis, from nucleoside diphosphate and ATP (Mishra et al., 2015). This activation of ATP production needed for energy, but also for DNA synthesis, could have contributed to the shorter infectious cycle observed *in vitro* for the attenuated strains (4 days after infection), with intensive DNA replication beginning quickly after infection of new host cells (Marcelino et al., 2015; this study for the Senegal strain). This result is consistent with the economic game theory proposed by Tago and Meyer to explain the attenuation mechanism of obligate intracellular bacteria (Tago and Meyer, 2016). In fact, the authors explain that, in *in vitro* conditions, bacteria decrease the expression of genes related to virulence and, in parallel, increase the expression of genes related to metabolism, which results in a better fitness and a shorter development cycle.

Interestingly, we observed a higher proportion of upregulated genes involved in coenzymes, carbohydrate, amino acid and inorganic ion transport and metabolism for the virulent Senegal strain. For this strain, lysis of host cells was observed at 6 days post-infection, whereas the virulent Gardel strain with a higher number of passages displays a lysis of host cells at 5 days post-infection. Replicates of virulent Senegal used in this study had a small number of passages *in vitro* (fewer than ten passages). This result reflects the progressive adaptation of *E. ruminantium* to the *in vitro* culture.

Like many intracellular pathogens, *E. ruminantium* seems to be able to fight efficiently against osmotic and oxidative stresses generated by host cells. Osmotic and oxidative stresses are key methods by which mammalian infected cells kill bacteria. Thioredoxin and Glutaredoxin are the key proteins that fight oxidative stress (Holmgren, 1989; Bjur et al., 2006). Genes encoding for glutaredoxin were found upregulated in attenuated Senegal. The upregulation of *grxC2* was also reported for *Rickettsia conorii* infected eschars (Renesto et al., 2008). It seems that attenuated strains activate a greater number of mechanisms against oxidative stress. To fight osmotic stress, intracellular bacteria have systems of transport or synthesis of osmoprotectants (or compatible solutes) that maintain homeostasis through their accumulation or rejection in the cytoplasm (Roessler and Müller, 2001). Both Senegal strains had differential expression of genes involved in fighting osmotic stress. Several genes were upregulated for each strain indicating a different activation of pathways between attenuated and virulent Senegal. For example, *proP*, which encodes for a proline/betaine transporter, was found upregulated for virulent Senegal. Proline and betaine are two osmoprotectants that work to overcome the inhibitory effects of hyperosmolarity. This gene was also found upregulated in *R. conorii* (Renesto et al., 2008).

For attenuated Senegal, four genes (*nuoA, nuoH, nuoK*, and *nuoL*) encoding for proteins of NADH-quinone oxidoreductase complex, were upregulated. This complex is usually involved in energy production, but it can help to maintain homeostasis as observed in *R. conorii* (Renesto et al., 2008). For the Gardel strains, there were no genes differentially expressed and involved in fighting osmotic stress. This result suggests that the virulent and attenuated Gardel strains could use common mechanisms to fight osmotic stress with the activation of similar genes.

Genes encoding for Map1-related proteins were exclusively upregulated in the virulent strains. However, *map* gene sequences were not different between the virulent and attenuated strains, which could explain the downregulation of *map1*-family gene expression (data not shown). The modulation of gene expression is probably due to mutations within unknown *map1* regulatory regions. These proteins are major antigenic proteins and in the case of Map1 induce a strong humoral response, which is not protective. Previous studies shown that *map1*-family genes were differentially expressed in host or vector cells suggesting that they are involved in the adaptation of *E. ruminantium* to its host or vector (Postigo et al., 2008). The upregulated expression of genes encoding for Map1-related proteins in the virulent strains suggests that they could induce a non-protective immune responses. We hypothesized that Map1-family proteins may play a role in luring the immune system. For the four strains, many genes encoding for hypothetical membrane proteins, as well as genes involved in posttranslational modifications and cell wall/membrane/envelope biogenesis, were upregulated. These results suggest membrane reorganization to escape immune response, as described previously for *Chlamydia trachomatis* (Nicholson et al., 2003) and *Rickettsia prowazekii* (Bechah et al., 2010). The lower expression of *map1*-family genes in the attenuated strains and the upregulation of other genes encoding for hypothetical membrane proteins could be important in inducing efficient immune responses. These hypothetical membrane proteins should be functionally characterized and could be potential candidates for vaccine development.

For example, CDS_05140 (hmp12) was one of the most upregulated genes in both attenuated strains. This gene encodes for a proteoform porin located in the outer membrane of *E. ruminantium* (Marcelino et al., 2015). Further studies are required to characterize the role of the protein encoded by CDS_05140 (hmp12), and it could be a potential candidate for vaccine development. Moreover, the function of two another hypothetical membrane proteins unique to *E. ruminantium* could also be studied: CDS_03690 (hmp9), which was upregulated for both attenuated strains, and CDS_04750 (hmp10), which was upregulated for both virulent strains.

In conclusion, the comparison of global transcriptomes of virulent and attenuated strains suggested a reorganization of the membrane of *E. ruminantium*. The upregulated membrane proteins expressed in the attenuated strains could be vaccine candidates against heartwater, and their function will be further studied.

In fact, genes encoding for Map1 proteins were found to be downregulated in both attenuated strains, suggesting that these proteins have a specific role in *E. ruminantium* pathogenesis. For both the virulent and attenuated strains, most of the upregulated genes encoding hypothetical membrane proteins were different between Gardel and Senegal, indicating the possible modification of membrane structures. These gene expression modifications could induce either unprotective or protective immune responses, highlighting the importance of studying these proteins.

## Author contributions

BM, DM, NV, and TL: conceived and designed the experiments; LP and KL: performed the experiments; LP, BM, KL, DM, and NV: analyzed the data; LP and NV: wrote the paper. All authors reviewed the manuscript.

### Conflict of interest statement

The authors declare that the research was conducted in the absence of any commercial or financial relationships that could be construed as a potential conflict of interest.
