# Supplementary material for: Comparative Transcriptome Profiling of Virulent and Attenuated Ehrlichia ruminantium Strains Highlighted Strong Regulation of map1- and Metabolism Related Genes
Source: Front Cell Infect Microbiol. 2018 May 15;8:153. doi: 10.3389/fcimb.2018.00153 (PMC5962694; doi:10.3389/fcimb.2018.00153)
Supplement: Supplementary file 1 [file Table_1.DOCX]

**S1 Table.** Primers sequences for *map1*-related genes qPCR validation

| Gene I.D. | Primer Name | Primer Sequence | Product Size (b.p.) |
| --- | --- | --- | --- |
| ERGA_CDS_09030 | *map1-12* F Gardel | AGTCCTCTAGGGATTGGTGGAGC | 163 |
|  | *map1-12* R Gardel | TGGTGAAGATGACTTGGTGTGGACT |  |
|  | *map1-12* F Senegal | AGAGTTAGAAGTATTTTATGAACA | 120 |
|  | *map1-12* R Senegal | AAATAAGTGATGGTGAGGAT |  |
| ERGA_CDS_09050 | *map1-10* F | TGTGCACAGGAATTGGTGGAG | 224 |
|  | *map1-10* R | TCCTTAACCCGACTTCGCTACC |  |
| ERGA_CDS_09060 | *map1-9* F | TGCTTTAGTCCGCAACAAAGATGA | 195 |
|  | *map1-9* R | TCCGCCAATTCCTAGACATGCG |  |
| ERGA_CDS_09090 | *map1-6* F | ATACACCAACATTCCAGAACA | 119 |
|  | *map1-6* R | CAGGGATTTCTGCATCGA |  |
| ERGA_CDS_09110 | *map1-4* F | TTGCACCAATTTCAGCACCA | 153 |
|  | *map1-4* R | AGCTTATTTGCAGATGGGTACT |  |
| ERGA_CDS_09120 | *map1-3* F | CTGTTATGATTAATGGATGTCATGA | 138 |
|  | *map1-3* R | CCAATTTTGCCTTGATAAGC |  |
| ERGA_CDS_09130 | *map1-2* F | CGATGCATTACACCTAAAACTTGCC | 162 |
|  | *map1-2* R | TGGGGTGTTATTCAGATCAGCAAC |  |
